# Supplementary figures and images for: Optimality of Mutation and Selection in Germinal Centers
Source: PLoS Comput Biol. 2010 Jun 3;6(6):e1000800. doi: 10.1371/journal.pcbi.1000800 (PMC2880589; doi:10.1371/journal.pcbi.1000800)

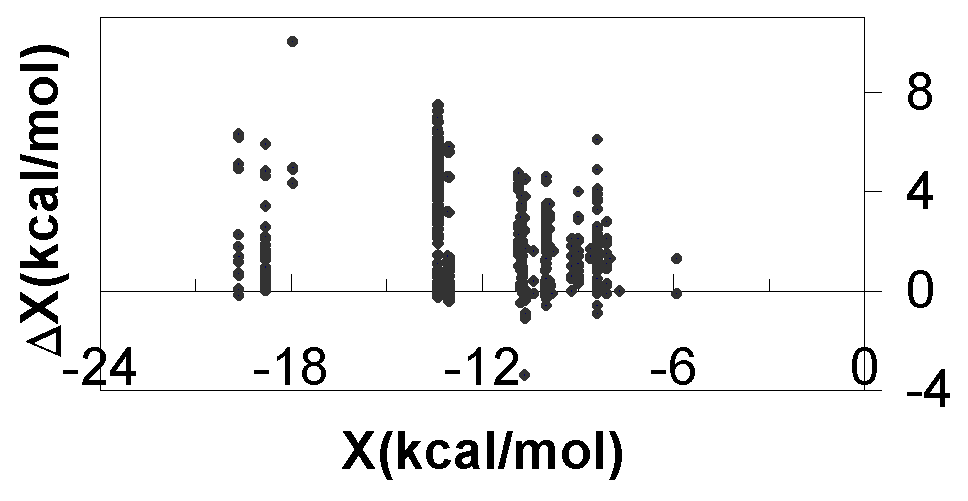

Supplement: Figure S1 — The scatter plot of affinity X and the change of affinity ΔX from PINT database, which does not show significant correlation. (0.05 MB TIF) [file pcbi.1000800.s001.tif]

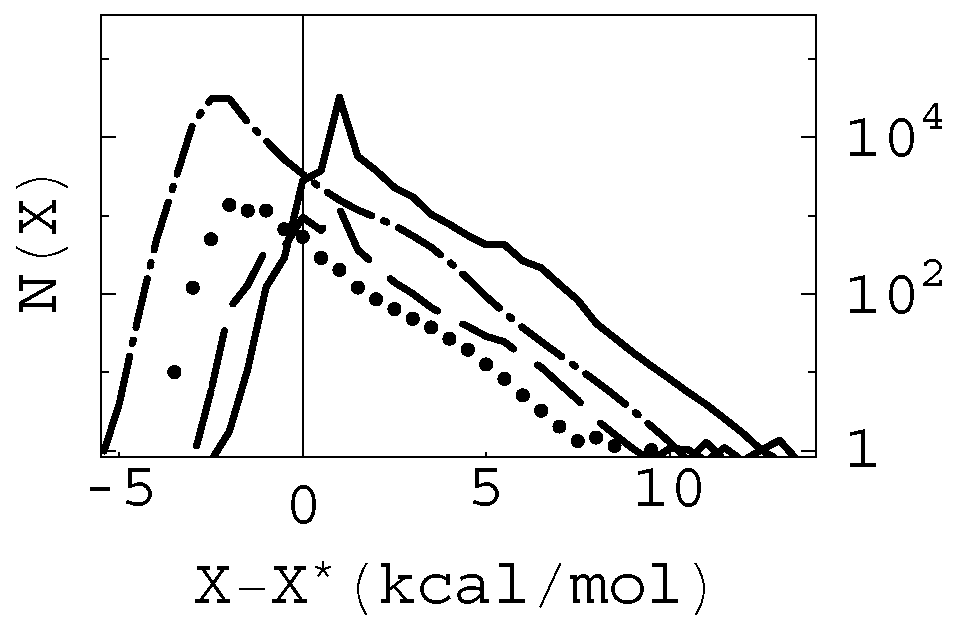

Supplement: Figure S2 — Affinity distribution in the population N(X,t) at t = 3 (solid), 6 (dashed), 9 (dotted), and 12 (dash-dotted) days, starting from germline affinity Xin = X *+1kcal/mol or Kain/Ka * = 0.18, with initial population N 0 = 106, selection strength b = 0.7/day(kcal/mol) and effective mutation rate mtotal = 2.8/day. The average affinity improves with time; while population size shrinks then grows. (0.06 MB TIF) [file pcbi.1000800.s002.tif]

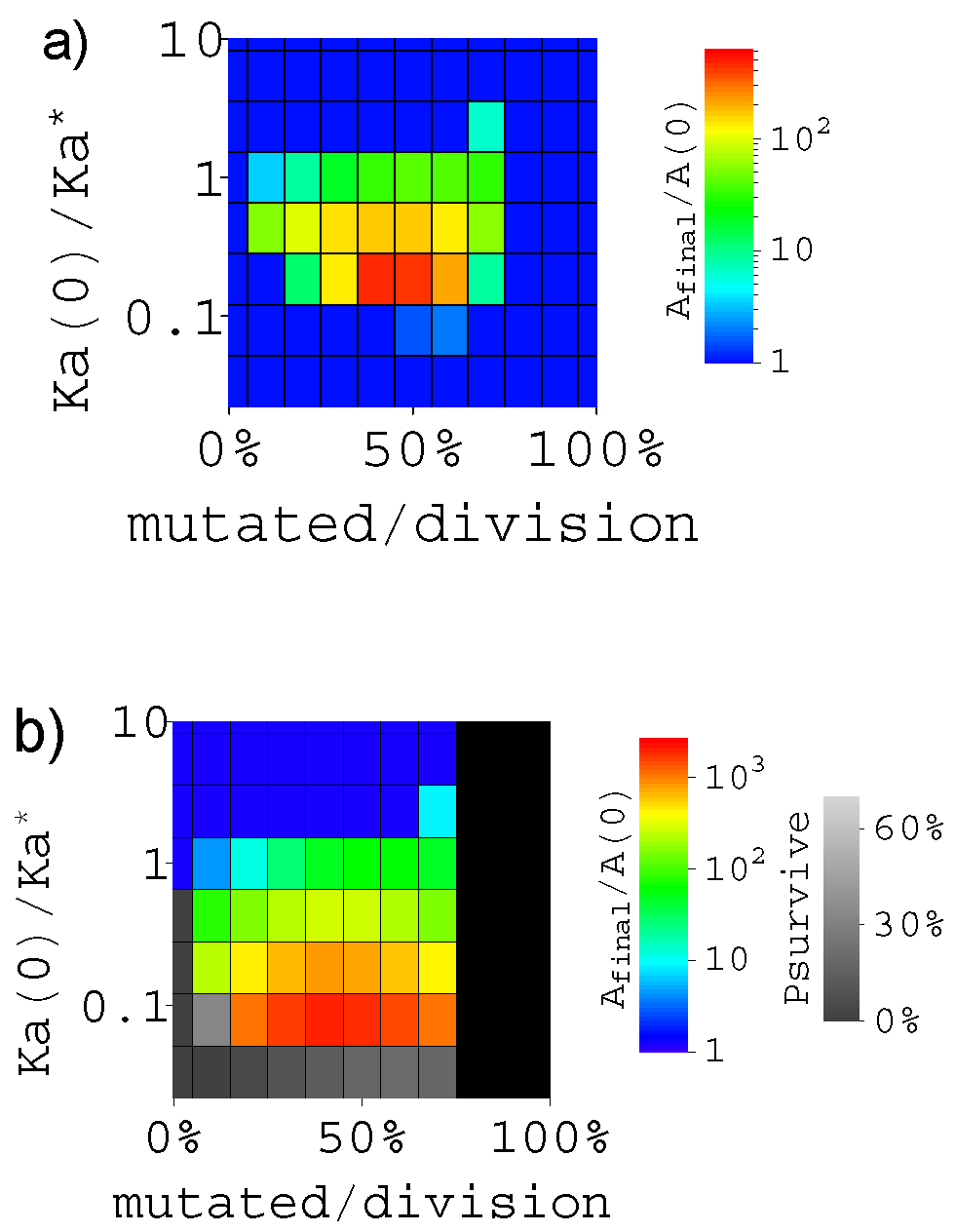

Supplement: Figure S3 — Optimization of b and m. The color indicates the improvement of total affinity. Here different initial affinities are tried for each mutation rate and b, and the one which gives largest affinity improvement is chosen. b = 1.2/day/(kcal/mol) is the global optimal selection strength. A minor local peak at b = 1.2/day/(kcal/mol) might be an artifact due to discrete (rather than continuous) choices of initial affinity values. (0.21 MB TIF) [file pcbi.1000800.s003.tif]

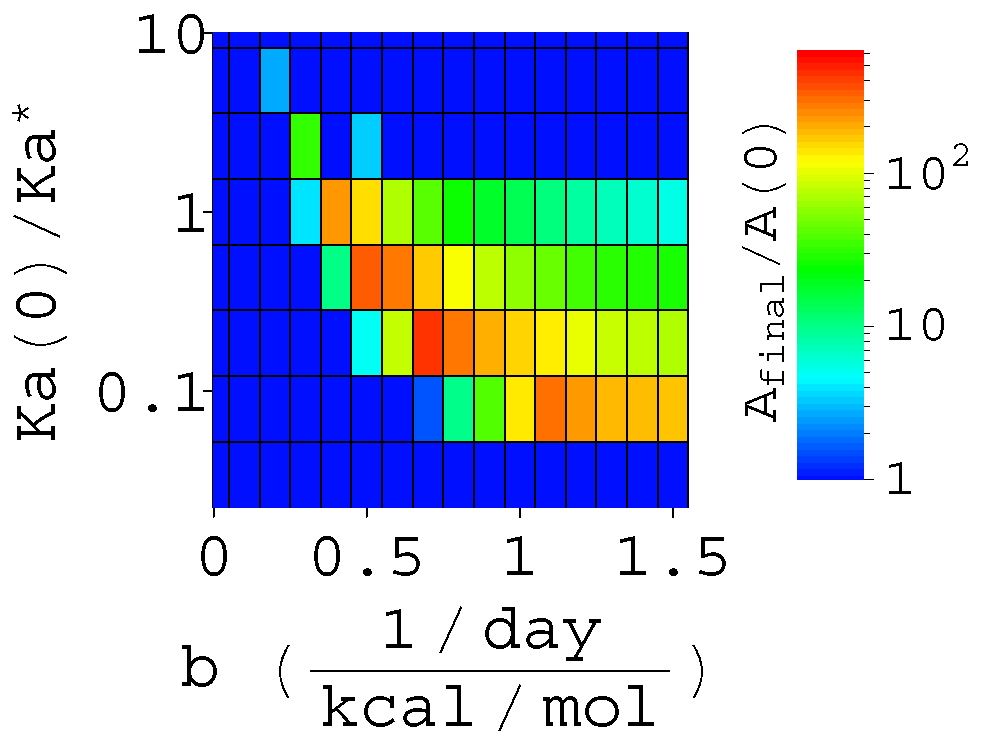

Supplement: Figure S4 — The improvement of total affinity as a function of selection strength and initial affinity. Here mutation rate is chosen as the optimum value, i.e. m = 0.55/day/gene or 50% mutated daughter cells. (0.16 MB TIF) [file pcbi.1000800.s004.tif]
