# Supplementary material for: Optimality of Mutation and Selection in Germinal Centers
Source: PLoS Comput Biol. 2010 Jun 3;6(6):e1000800. doi: 10.1371/journal.pcbi.1000800 (PMC2880589; doi:10.1371/journal.pcbi.1000800)
Supplement: Text S1 — This file contains supporting text. (0.07 MB DOC) [file pcbi.1000800.s005.doc]

**Text S1**

**Affinity change upon mutations:** From the PINT (protein interaction) database[1], the change of affinity upon mutations does not have obvious correlation with the affinity before mutations (Figure S1). The fraction of beneficial mutations has no significant dependence on the affinity, especially for the typical range of affinity maturation $10^{6}M^{-1}<Ka<10^{9}M^{-1}$ or $-12kcal/mol<X<-8kcal/mol$. The detrimental mutations in Figure 1 can be fitted by $W(\Delta X)=0.48\exp[-\Delta X/(1.87kcal/mol)]$, while the data of beneficial mutations can be fitted by $W(\Delta X)=0.18\exp[-\Delta X/(0.43kcal/mol)]$.

**Survival probability:** If the initial B cell population is too small, the population might not pass through the bottleneck, but rather becomes extinct. To calculate the survival probability of the whole population numerically, we start from an initial B cell with weak affinity *X*, corresponding to a death rate $\left|B(X)\right|$ larger than the replication rate $r-0.3m_{total}$. The initial cell and its descendants could either die or replication and/or mutate with some well defined probabilities. By collecting the probability distribution of each cell’s fate, we numerically calculate the probability for some descendants to reach a strong affinity $X*-10h$. In this way we find the probability *p* for some descendants of the initial B cell with affinity X to eventually survive, and use *p* to find the expected probability for the system of $10^{6}$ initial B cells to survive rather than become extinct.

**Gradual improvement of affinity:** We can monitor the distribution of affinity at different moments (Figure S2). The population starts from identical weak affinity B cells $X_{in}=X*+1kcal/mol$ or $Ka_{in}/Ka*=0.18$, and has an initial population decrease; but B cells with stronger affinities gradually emerge, and become dominant due to selection, and the population size grows thereafter. The continuous improvement of affinity is supported by experiments[2].

**Affinity Improvement as a function of selection strength:** We look for the optimal values of mutation rate *m*, selection strength *b* and initial binding level $X_{in}-X*$ that maximize affinity improvement for the case of fast B cell migration between GCs (i.e. $N_{0}=10^{6}$ initial B cells). The variables in Figure 3a are *m* and $X_{in}-X*$, and here we plot the improvement of affinity in the plane of *b* and *m* (Figure S3), and in the plane of *b* and $X_{in}-X*$ (Figure S4). At each given value of *b* and *m* in Figure S3, different initial affinities $X_{in}-X*=0$, 0.5, 1, 1.5, and 2kcal/mol are calculated, and the initial affinity resulting in the strongest affinity improvement is chosen. To maximize the affinity improvement, the optimal mutation rate is at 50% daughter cells mutated at each replication, and the optimal selection strength is b=0.7/day/(kcal/mol).

**References**

1. Kumar MD, Gromiha MM (2006) PINT: Protein-protein Interactions Thermodynamic Database. Nucleic Acids Res 34: D195-198.

2. Kocks C, Rajewsky K (1988) Stepwise intraclonal maturation of antibody affinity through somatic hypermutation. Proc Natl Acad Sci U S A 85: 8206-8210.

**Figure Captions**

Figure S1: The scatter plot of affinity X and the change of affinity $\Delta X$ from PINT database, which does not show significant correlation.

Figure S2: Affinity distribution in the population $N(X,t)$ at t=3 (solid), 6 (dashed), 9 (dotted), and 12 (dash-dotted) days, starting from germline affinity $X_{in}=X*+1kcal/mol$ or $Ka_{in}/Ka*=0.18$, with initial population $N_{0}=10^{6}$, selection strength $b=0.7/day(kcal/mol)$ and effective mutation rate$m_{total}=2.8/day$. The average affinity improves with time; while population size shrinks then grows.

Figure S3: Optimization of *b* and *m*. The color indicates the improvement of total affinity. Here different initial affinities are tried for each mutation rate and b, and the one which gives largest affinity improvement is chosen. b=1.2/day/(kcal/mol) is the global optimal selection strength. A minor local peak at b=1.2/day/(kcal/mol) might be an artifact due to discrete (rather than continuous) choices of initial affinity values.

Figure S4: The improvement of total affinity as a function of selection strength and initial affinity. Here mutation rate is chosen as the optimum value, i.e. m=0.55/day/gene or 50% mutated daughter cells.
